# Cybersecurity in Hospitals: A Systematic, Organizational Perspective

**DOI:** 10.2196/10059

**Published:** 2018-05-28

**Authors:** Mohammad S Jalali, Jessica P Kaiser

**Affiliations:** ^1^ MIT Sloan School of Management Massachusetts Institute of Technology Cambridge, MA United States

**Keywords:** cybersecurity, hospitals, organizational models, computer simulation

## Abstract

**Background:**

Cybersecurity incidents are a growing threat to the health care industry in general and hospitals in particular. The health care industry has lagged behind other industries in protecting its main stakeholder (ie, patients), and now hospitals must invest considerable capital and effort in protecting their systems. However, this is easier said than done because hospitals are extraordinarily technology-saturated, complex organizations with high end point complexity, internal politics, and regulatory pressures.

**Objective:**

The purpose of this study was to develop a systematic and organizational perspective for studying (1) the dynamics of cybersecurity capability development at hospitals and (2) how these internal organizational dynamics interact to form a system of hospital cybersecurity in the United States.

**Methods:**

We conducted interviews with hospital chief information officers, chief information security officers, and health care cybersecurity experts; analyzed the interview data; and developed a system dynamics model that unravels the mechanisms by which hospitals build cybersecurity capabilities. We then use simulation analysis to examine how changes to variables within the model affect the likelihood of cyberattacks across both individual hospitals and a system of hospitals.

**Results:**

We discuss several key mechanisms that hospitals use to reduce the likelihood of cybercriminal activity. The variable that most influences the risk of cyberattack in a hospital is end point complexity, followed by internal stakeholder alignment. Although resource availability is important in fueling efforts to close cybersecurity capability gaps, low levels of resources could be compensated for by setting a high target level of cybersecurity.

**Conclusions:**

To enhance cybersecurity capabilities at hospitals, the main focus of chief information officers and chief information security officers should be on reducing end point complexity and improving internal stakeholder alignment. These strategies can solve cybersecurity problems more effectively than blindly pursuing more resources. On a macro level, the cyber vulnerability of a country’s hospital infrastructure is affected by the vulnerabilities of all individual hospitals. In this large system, reducing variation in resource availability makes the whole system less vulnerable—a few hospitals with low resources for cybersecurity threaten the entire infrastructure of health care. In other words, hospitals need to move forward together to make the industry less attractive to cybercriminals. Moreover, although compliance is essential, it does not equal security. Hospitals should set their target level of cybersecurity beyond the requirements of current regulations and policies. As of today, policies mostly address data privacy, not data security. Thus, policy makers need to introduce policies that not only raise the target level of cybersecurity capabilities but also reduce the variability in resource availability across the entire health care system.

## Introduction

Health care data breaches are a growing threat to the health care industry, causing not only data loss and monetary theft but also attacks on medical devices and infrastructure [1]. Hospital data security breaches in particular have the potential to cost a single hospital as much as US $7 million, including fines, litigation, and damaged reputation [2]. A data breach has a combined estimated effect on the health care industry of about US $6 billion [3]. Meanwhile, the health care industry lags behind other industries in securing its data, and in response, health care organizations must invest considerable capital and effort in protecting their systems [4].

However, this is easier said than done, given the complexity of health care organizations. Hospitals are extraordinarily complex organizations with many typical organizational characteristics dialed up or down to extremes [5] such as

- Technology saturated environment: similar to other organizations, they struggle to manage an array of devices ranging from legacy information technology (IT) to connected medical devices; unlike other organizations, they have orders of magnitude more of them, procured not by a single IT department but purchased ad hoc by clinicians, or given for free by medical device companies [6].
- Internal politics: they deal with the same internal politics that other large organizations do but complicated by the complexity of functions contained within the organization: finance, IT, and human resources, just like other organizations; unlike other organizations, they also must support radiology, cardiology, and pediatrics among others [5]. The degree of specialization is high. Each department requires totally different equipment, caters to different patient needs, has different workflows, and employs a highly specialized labor force that requires years to train.
- Regulatory pressures: similar to other organizations, they must abide by the regulations imposed on them by state and federal government; but in the United States, health care data is considered to be particularly sensitive, and thus, is protected under additional specific data protection laws [7].
- Patient-centered care: like all organizations in the United States, hospitals care about their ability to generate positive net revenue for survival, but unlike other organizations, their first mission is to care for their patients, even when they are for-profit [8].

It is interesting to consider what the systemic effect of these characteristics might be on a single hospital’s ability to remain robust to cyber breaches. But now consider the range of possible differences among these entities, eg, a rural community hospital has dramatically different priorities than a large, urban research hospital. Specific to IT, outsourcing services is more common in smaller or more rural hospitals, with transcription services being the most commonly outsourced function [9,10]. The decision to outsource interacts with the tendency of these hospitals to make symbolic rather than substantive IT security investments—see Angst and Kelley [11] for more discussion.

Furthermore, significant variability in cybersecurity as a priority has been observed throughout the hospital industry—in the United States, 70% of hospital boards include cybersecurity in their risk management oversight, and only 37% of hospitals perform annual incident response exercises [12]. Similar vulnerabilities in hospitals are also observed in other countries [13-16]. Specifically, pressure from the board of directors appears to be essential in creating substantive cyber resiliency, as research shows that hospital management support is essential for user compliance with information security policies, which in turn are written by health care IT security professionals [17,18].

The importance and complexity of cybersecurity capability development at hospitals raise critical questions: how do the inter- and intradynamics of hospitals interact to form a system of hospital cybersecurity in the United States? Does this leave the health care infrastructure of the United States vulnerable as a whole? As data interoperability becomes an imperative, driven by Affordable Care Act requirements and payment reform, will hospitals with lower cyber capabilities leave all patients vulnerable?

To answer these questions, we interviewed chief information officers (CIOs), chief information security officers (CISOs), and health care cybersecurity experts at hospitals and developed a system dynamics model to study the dynamics of implementation and maintenance of cybersecurity capabilities in hospitals.

This study helps health care leaders reduce hospital vulnerabilities by detailing the outcomes resulting from strategic decisions of cybersecurity development. It also aids cybersecurity professionals in understanding the complexities of cybersecurity capability development in hospitals.

## Methods

To develop our model, we conducted semistructured interviews with 19 cybersecurity professionals, primarily in the United States. Interview subjects were contacted by email and volunteered to participate in a 45-min interview on cybersecurity capabilities at their hospitals, noting that all interviews would be stripped of identifying details. Given previous research showing significant differences in capability development driven by size, orientation, and urbanicity, we strove to obtain diverse candidates that represented dimensions previously shown to matter in capability development. To minimize biases (eg, interviewer bias and confirmation bias), we followed standards for semistructured interviews [19], specifically, we ensured that (1) Interviewers were educated to maintain a neutral attitude and avoid suggesting answers or making judgments and (2) The predefined questions had a neutral language and did not include suggestive words. Interviewees included (1) C-level executives who actively participated in the strategies of hospitals, (2) Operationally focused information security professionals, who typically had titles such as information security specialist in hospitals, and (3) Software vendors and consultants with a privacy and security focus, who specialized in the health care industry (see Multimedia Appendix 1 for more information).

Following standards for inductive and generative coding [20], the interview data were coded to extract variables and common themes related to cybersecurity capability development in health care organizations. Coding of the interviews was conducted by the authors—any disagreements or concerns about the extracted data were discussed among the authors until consensus was reached. Coding helped in learning the mechanisms of capability development through identifying key variables and relationships among the variables. For example, “efforts for filling out gaps” (between the actual and desired level of cybersecurity) and “internal stakeholder alignment and resource availability” are two variables extracted from the interviews, and the relationship between these two variables was that “internal stakeholder alignment and resource availability” had a positive causality effect on “efforts for filling out gaps.”

Following best practices for analyzing qualitative data to build system dynamics models [21], the emerging relationships among the extracted variables were then integrated into an evolving causal loop diagram, which embedded the key relevant mechanisms important for understanding how the capabilities were built and then eroded—see the next section for the description of the model. We used system dynamics modeling, which is a potential tool to understand the complexity of a sociotechnical system [22-25]; in this case, the system of cybersecurity capabilities in hospitals.

## Results

### Main Findings

Cybersecurity capabilities include a variety of programs, behaviors, and technologies that a hospital employs to improve cyber resiliency. Not all of these capabilities are self-sustaining, and they may erode over time. If properly adopted, implemented, and maintained, however, they will have a positive impact on the hospital’s ability to be resilient to cyberattacks. Stock and flow variables are key tools in system dynamics to present this mechanism.

**Figure 1:**
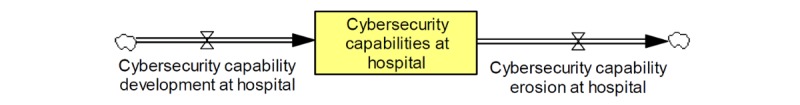
Stock and flow diagram of hospital cybersecurity capabilities.

Figure 1 shows the simple core of our model, including stock and flow variables. A stock variable (eg, “cybersecurity capabilities at hospital” in Figure 1) presents accumulations such as the number of implemented programs, behaviors, and technologies, which represent a hospital’s cybersecurity capabilities. A flow presents the rate at which the stock changes (see the inflow and outflow variables in Figure 1). The inflow is the capability development rate or the rate at which capabilities are added to the existing stock. The outflow is the capability erosion rate or the rate at which capabilities are removed from the existing stock.

Our interviews uncovered five major themes that described the dynamics of the cyber capability development in hospitals: uncertainty in resource availability, external pressures, end point complexity, internal stakeholder alignment, and cybercriminal activity. In this section, we expand on each theme by constructing our model and using quotes from interview data to show how our subjects described each mechanism behaving.

### Uncertainty in Resource Availability

Current business trends in health care were a factor in our interview subject’s minds and produced the most uncertainty with regards to how they affected cybersecurity capability development through resources available to the information security team. Two subjects stated the following:

With all the financial pressures on hospitals right now, [cybersecurity] is not their big concern. The biggest driver is available funding.

A larger organization can hire more security admins and come up with more purchases, or different products, or come up with more protocols, or create more tools that they need to monitor the network more closely. For an organization that either has fewer resources or chooses not to invest in that, they are more likely to look at hosted solutions.

The two main issues affecting resource availability were net revenues and talent availability. For most interviewees, net revenues were perceived to be declining, driven by flat revenues and increasing operating expenses. For organizations with declining net revenues, outsourcing IT to an organization with more expertise was an effort to increase resource availability to undertake more efforts to close cybersecurity gaps. Some of our interview subjects worked at organizations that were financially healthy enough to fund the development of purely internal solutions; however, the majority did not. Two subjects stated the following:

We do not develop solutions, because we are too small...So we need to focus on existing solutions.

I am increasingly looking at technology as a solution for process problems, or issues that I see in my center...I can’t justify every dollar that I’m going to spend on it if it’s going to give me more in return or value. But what I do know is that to make the same kind of changes in one big swoop, would require incredible patience, diplomacy, personal political capital. I can pay $20k for it and potentially other problems get solved. Or, I can pull up my sleeves and make the same changes on my own, but I would almost certainly have a Xanax dependency at the end of it.

For those who worked at organizations healthy enough to fund internal development, subjects were split as to whether self-hosting and internal development increased resource availability. On one hand, some felt that owning IT policies themselves gave them finer control over how to allocate resources in their efforts to close cybersecurity gaps. On the other hand, some felt that outsourcing security operations to a firm such as Microsoft via purchases of their cloud products simultaneously allowed them to do more with fewer resources and also tacitly allowed them to pay less attention to cybersecurity, thereby introducing an entirely new set of risks.

Three subjects stated the following:

I do think there are areas where we can do a little more, because it’s easy to outsource those responsibilities in a sense. So it’s easy to point the finger and say we depend on these people for that.

There’s a real imperative to go to cloud-hosted services and procure those services. There’s a different stack of security issues to think about if you’re purchasing subscription services if you’re doing it yourself.

For an organization that either has fewer resources or chooses not to invest in [cybersecurity], they are more likely to look at hosted solutions...The push to get more things in someone else’s cloud will help those organizations standardize those security practices.

For the stand-alone organizations who were not part of a larger organization or part of an urban environment, urbanization among the US population had affected their ability to hire security professionals, who mostly live in urban centers. Furthermore, because the populations of these urban centers are growing, the patient populations that those hospitals serve have grown as well. This trend had a strong impact to resource availability for hospitals outside of larger urban areas. Two subjects stated the following:

How many trained security professionals are there in South Dakota?

Healthcare doesn’t get paid very much, so revenue doesn’t go towards cybersecurity. When in banking, I would have had 25 employees at an organization of this size.

**Figure 2:**
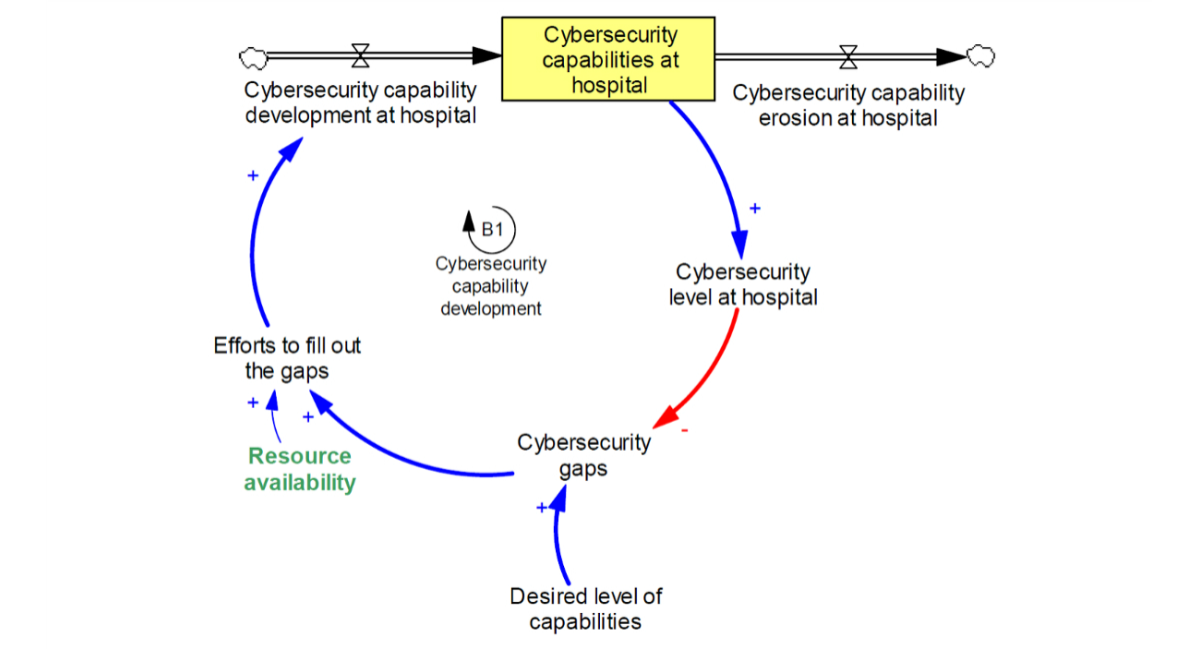
Cybersecurity capability development with a balancing feedback loop.

Our interviews suggested that a hospital with declining net revenues would also typically have troubles attracting talent as well. Thus, we described these two concerns in the model through the single variable “resource availability.” Whether those resources were “admins, purchases, products, protocols, or tools”—as described by the interview subject above—these were the essential building blocks that allowed a hospital to make efforts to increase the capability development rate, thereby increasing the stock of capabilities at the hospital. This, in turn, would increase the cybersecurity level at the hospital, which would decrease the gaps between the actual and desired level of cybersecurity. If the gaps decreased, then so would efforts to fill out those gaps. Figure 2 presents the efforts to develop cybersecurity capabilities as driven by the variable of resource availability. Feedback loop B1 presents a balancing feedback loop that stabilizes the system (ie, filling out cybersecurity gaps) by moving it toward the desired goal.

### External Pressures

In the previous section, we describe the cybersecurity level at the hospital, as influenced by the stock of cybersecurity capabilities. This cybersecurity level also drives the vulnerabilities that cybercriminals can exploit, and, if successfully exploited, the hacks and breaches which then affect hospitals. Subjects spoke of the many ways that these successful breaches translated into pressures to develop stronger capabilities.

Our interview subjects often used a successful cybercriminal exploit at another hospital to stoke higher pressure for cybersecurity capabilities by bringing the consequences of that exploit to the attention of their board or managers. They were typically speaking of the pressures imposed by the public and the media and those imposed by Health Insurance Portability and Accountability Act (HIPAA) and related regulation and, more recently, from the US Food and Drug Administration (FDA) in the arena of medical devices.

A major pressure to have stronger capabilities was the threat of a loss of public reputation. This fear is in part by design of existing regulation, which includes reporting requirements should a cyber breach occur. But the threat of a public or media backlash was real and weighed on our interview subject’s minds as a “sniff test” for whether they should be doing more to develop cybersecurity capabilities. It also speaks to the importance of the media in generating awareness around cyber threats, particularly for health care organizations. Two subjects stated the following:

Our culture wasn’t this way seven years ago...It also took bad things happening sometimes. Nothing affects change like someone making a mistake.

What Rahm [Emanuel] told us is let no emergency or crisis go unused...If you say [to the board], look Home Depot has just had this breach and this expense...Don’t you think we want to avoid being in the Boston Globe in a bad way?

There were also significant pressures to have stronger cybersecurity capabilities resulting from regulation. Typically, regulation was aimed at protecting privacy, not necessarily security; nonetheless, there was some overlap. Subjects at hospitals with more resources worked with both an internal and an external audit team to assess compliance; however, all hospitals worked with an external audit team as a regulatory requirement.

Subjects expressed a variety of views with regards to how helpful regulations were in producing good cyber hygiene, as illustrated in the following quotes:

I think the larger part of the regulatory requirements is absent of any alignment with cybersecurity. They’re largely focused purely on patient health and the patient experience of care, and...they’re largely divorced from cybersecurity.

You have to [follow regulation] because you need some common grounding and things to measure against and things to work to. It serves a good purpose.

Some felt that HIPAA created a floor of cyber capabilities that was helpful for small organizations, but not larger ones, as illustrated in the following quotes:

I think all kind of clichés about it, which is that it’s a floor, not a ceiling, and that you can be compliant but not secure. I think those are mainly right.

So we’re a small center, nobody expects me to have one FTE manager whose sole job is to walk around all day looking specifically for HIPAA violations. So that wouldn’t be a reasonable standard. What is reasonable is do we train people, do I as a leader emphasize it routinely. I don’t like the words minimum necessary, but it is kind of the minimum necessary needed to meet the intent [of eliminating data breaches].

Compliance is a low bar. I guarantee that little healthcare organizations and hospitals would do nothing [without regulation]. They would have a piece of paper on a shelf called their security policy. It’s needed as a backstop to get companies at least thinking about it. But being compliant does not solve the greater risk management problem.

Some felt that the pressures produced by HIPAA interacted with the target level of cybersecurity capabilities in such a way that the resultant desired level of cybersecurity capabilities encouraged hospitals to focus on the wrong things. One subject stated the following:

Clearly, HIPAA distorted the cybersecurity programs of large organizations a little bit. I treat compliance as a separate issue from security. Let’s make sure that we’re plausibly compliant and let’s build a program over actual security.

In particular, there was a belief that the focus on end-to-end messaging encryption was not as important for hospitals to focus on, as illustrated in the following quotes:

Say you have an electronic medical record that sits in the same lab, but the data gets transferred between the two systems. You can say this is very safe, because in order for the bad guy to find it, they’d have to go through so many layers that they’d have to be in the hospital anyway. Some auditors say it has to be encrypted anyway, which might increase complexity, time performance, and even data availability. The more complex your systems get, the slower they run, which affects availability.

A lot of healthcare organizations have been built around encrypted e-mail which to my mind don’t have real security benefits...You want to reduce the emails people send with private info, but people don’t spend a lot of time thinking about that.

Others mentioned other “best practice” security practices that are not mentioned in regulations but would help prevent data breaches, as illustrated in the following quote:

It’s impossible to have a good security without pentesting, without a very active threat hunting program. Those are the kind of things that we really emphasize. But they’re not generally contemplated in the general HIPAA regulatory regime.

And some felt that recent regulations, such as the Health Information Technology for Economic and Clinical Health Act (HITECH), were quite forward-looking and pushed their organization forward, whereas others did not, as illustrated in the following quotes:

It’s really HITECH rather than HIPAA [that we focus on compliance]. That was the whole Obama-era program that resulted in audits and compliance.

I think HITECH does go above and beyond. It puts out their technical controls. And it gives you a great starting base for talking to the business owner.

HITECH is just establishing subcontractors have to abide by HIPAA. It just means there’s more you have to follow. I’ve never heard HITECH being called out separately from HIPAA.

What is clear is that the process of external audit at least compels hospitals to adopt some cybersecurity standard (examples given were National Institute of Standards and Technology [NIST] 800-66, Control Objectives for Information and Related Technologies, and Information Technology Infrastructure Library) and try to follow it. On subject stated the following:

You need to set yourself around a common cybersecurity framework. It doesn’t have to be ISO or NIST, it could be a combo. It fits the general corporate culture as it is here and now.

Interview subjects stated that external auditors varied in the degree to which they demanded rigorous compliance to that standard but that the standard gave them a helpful tool in socializing good cybersecurity practices throughout the organization. Two subjects stated the following:

It’s a little confusing to be having been an auditor. You assess an organization against a set of known criteria. I see auditors doing less of that, and more taking freedoms and liberties.

We use NIST 800-66 or other NIST artifacts to decide what is the rubric for our risks and our risk mitigations. You kind of have to pick a rubric—whether it’s HITRUST [the Health Information Trust Alliance]...or COBIT [Control Objectives for Information and Related Technologies] or ITIL [Information Technology Infrastructure Library]. It is not sufficient for three people to sit at a table with a bunch of beer and decide the risks. Our boards and our auditors have asked us to adopt a standard framework, whether it’s Deloitte or PwC, so they can judge us against an objective framework of goodness. That’s truly essential for an org.

**Figure 3:**
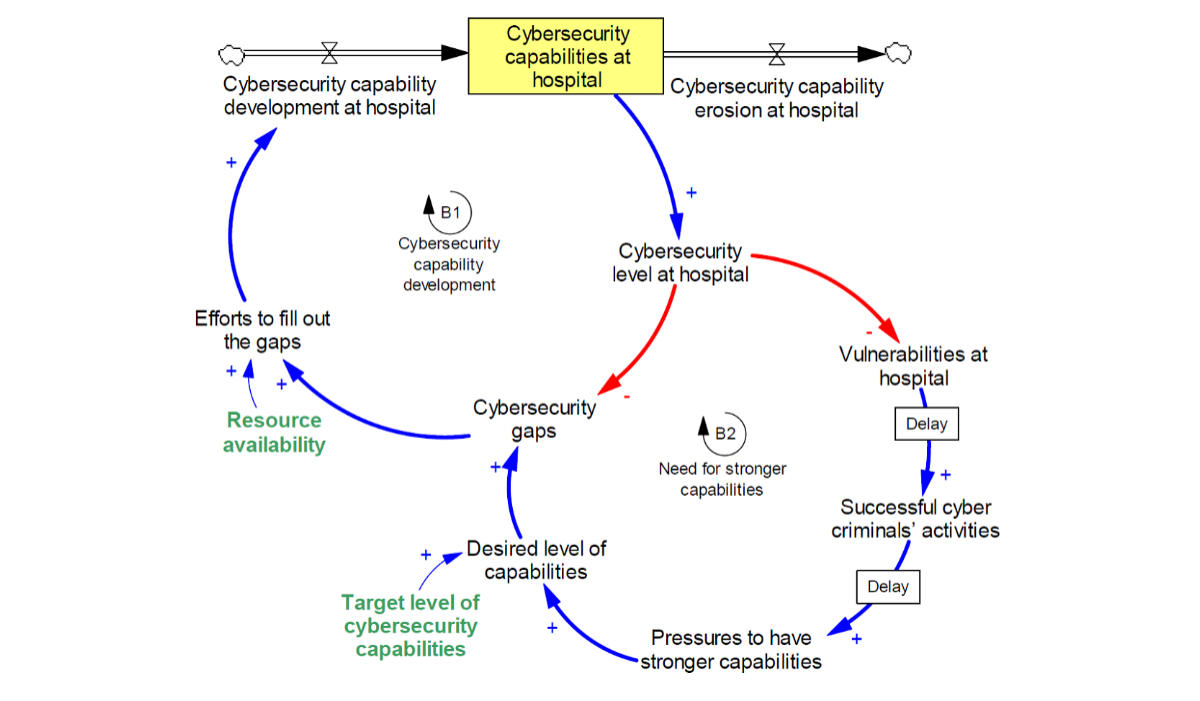
Balancing feedback loop of need for stronger capabilities.

These pressures (either internal or external), combined with the target level of cybersecurity capabilities, produce the hospital’s target level of capabilities. The target level and the desired level may be different (see Figure 3 which adds the feedback loop “need for stronger capabilities”). Loop B2 is a balancing loop, with delays in how quickly vulnerabilities and cybercriminal activities ultimately affect the pressures to have stronger capabilities.

### End Point Complexity

The theme explored by most interview subjects was that end point complexity made hospital cybersecurity capabilities unique. Like many organizations with a large employee base and a physical footprint, hospitals must manage the numerous devices used by both administrators, medical staff, patients, and their visitors to transact business, provide care, and pass time. One subject stated the following:

I have 8000 iPhones, 2000 Androids, 2000 iPads, and some Blackberries. What are the security implications of doctors and patients doing more transactions on BYOD [bring your own device] devices?

Unlike many organizations, the bring your own device (BYOD) challenge is compounded by a bevy of instrumentation and diagnostic equipment that may also present security risks. One subject stated the following:

In our environment, we have about 800 families of medical devices. Most organizations have two or three dozen SCADA systems...That’s an astonishingly high number. There’s no counterpart to that in education or finance.

In a competitive market, some medical device manufacturers also provide “free samples,” bypassing risk assessment and management processes. Although IT and information security (IS) teams have methods of determining unauthorized connections to their networks by unexpected devices, it adds another several points of vulnerability to their organization to contend with these devices. One subject stated the following:

In hospitals, the interesting stuff is there’s a whole underground procurement process whereby medical device vendors approach clinicians and give them lots of stuff for free that eventually makes its way on to our floors, and then a year later we get a bill for it. That’s a unique quality of working in a hospital.

Additionally, medical device manufacturers have historically not designed their products with security in mind. Interview subjects were optimistic that this might be shifting, as the FDA has waded into the regulation of the medical device market. However, they felt the process would be slow, as the FDA is slow to certify devices, creating a gap between regulation and practice that exposes patients to more risk. Three subjects stated the following:

We’re doing infusion pump management and we’re using a compromised Linux kernel. I can deliver a lethal dose and then back right out of it. We don’t even have checksums in those OS’s to do forensics.

The cybersecurity of your pump that sits next to the patient that programmatically determines when to pump medications into an IV [intravenous], the security of that device now needs to be a primary design decision. It needs to be a motivating factor in when you create that device. So the regulation that the FDA—the guidance—feels a little behind the times. But they’re catching up with the need to put out guidelines on these things.

It’s a highly regulated industry [by the] FDA. When you make a change to those systems, you have to go back and recertify...They end up not doing that and you end up with machines that can [be] breach[ed].

**Figure 4:**
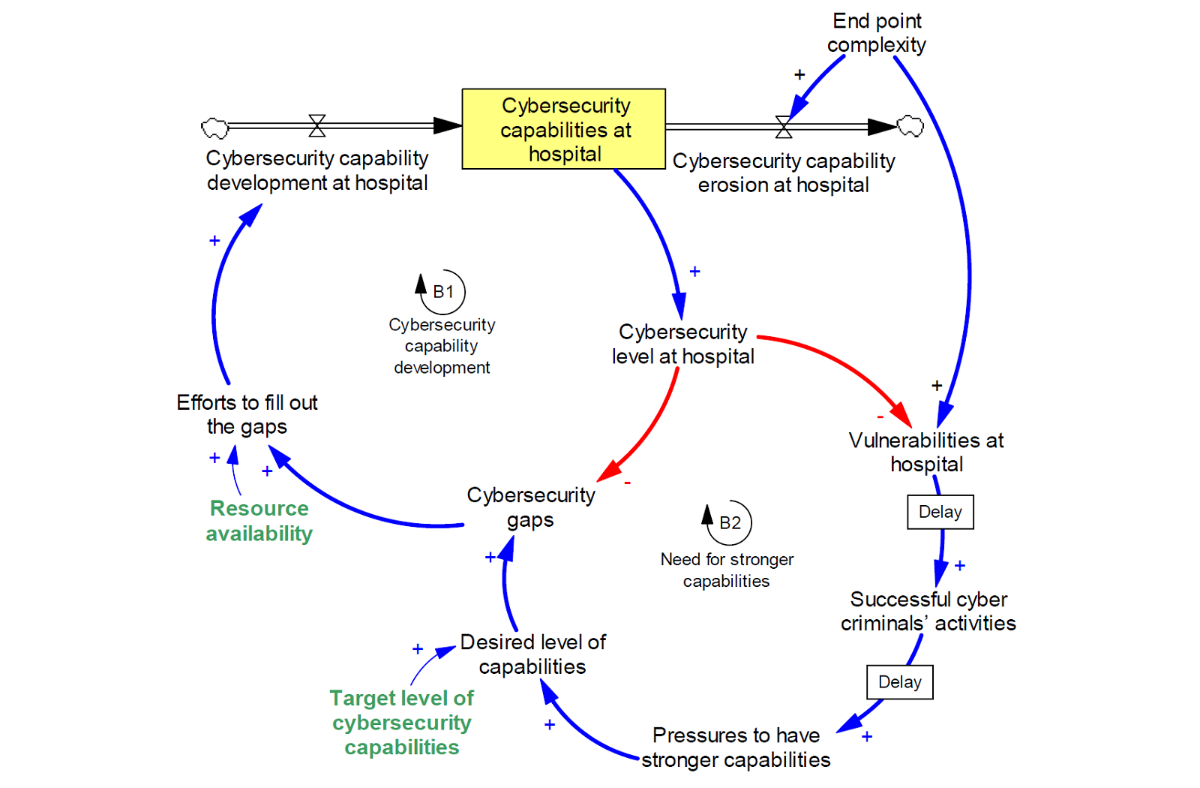
Addition of end point complexity to capability development model.

Thus, we add end point complexity to the model. As the end point complexity increases, it increases the vulnerabilities at the hospital. Also, with the increases of end point complexity, the ability of the organization to manage the security of each end point degrades. The result is that an increase in end point complexity subsequently increases the speed at which cybersecurity capabilities erode (see Figure 4 which adds end point complexity to the system).

### Internal Stakeholder Alignment

A major mechanism that our interview subjects described was of the complexity of internal stakeholder alignment—*There’s no single point of decision making*. Typically, the main stakeholders described were the CIO or CISO, the IS team (if one existed), the IT team, other C-level executives, the board of directors, and finally, the medical staff. Even in a small hospital with 100 beds, employee count will easily number in the hundreds.

First, the CIO and, if relevant, the CISO, had to develop alignment on strategic IT initiatives to bring those initiatives to the strategic planning process. All of our interview subjects described some level of friction if the IT and the IS resources were separated, as illustrated in the following quote:

Balancing how much should come from security and from general IT is one of the perennial problems that we deal with...That friction is always at the forefront when we do operational security.

Typically, IT advocated for the benefits of the technology, and the IS team had difficulty looking past the risks. They would come together to work out a compromise that adequately captured both of their viewpoints. Then, they would bring these perspectives to executive management during the planning process, using their organization’s preferred risk or value metrics to measure the impact of new or continued investments. One subject stated the following:

I make the decision[s on deploying new technologies] with the help of higher management. I present [options] to the board and get their approvals and address their concerns. The metrics that we use [to evaluate] are security values.

Our interview subjects who felt that top management was respective to their requests felt there were two overlapping reasons behind it: (1) there is clear evidence that underdevelopment of cybersecurity capabilities would result in a crisis for the hospital and (2) their board of directors also understood this and put pressure on the top level board to include cybersecurity as part of the hospital’s core strategy, as illustrated in the following quotes:

Boards and other fiduciary individuals have said, we need to buy down the risk. This isn’t some techie thing, this is risk to your business. What happens if doctors can’t do dictation? Am I going to remember everything I put down two days later? If we can’t code, we can’t bill, if you can’t bill, you don’t have revenue. If you’re not for profit, you run out of resources. It’s a long stretch but they’re starting to get it.

I get calls at my desk about this from board members...they are very engaged and very nervous. I have a decent level of traction despite that I’m many tiers down.

The board contains people whose companies have had material harm done to them because of cybersecurity breaches. So when the board comes to the senior executive team, or else you’ll be looking for a new job. That’s probably the most significant motivator—it’s some higher force than the senior management raising the issue.

It seemed that the threat of regulatory fines served to produce stakeholder alignment, to some extent, with IT, top management, and the board of directors more easily, as illustrated in the following quote:

It’s a board level issue because of the reputational issue and fines that occur in a breach. The average cost of a breach is $300 per patient breached. When you look at the legal fees, forensics, media management, fines from Office of Civil Rights—the board says this is an existential issue. There are only a couple of ways we can fire the CEO and loss of reputation is a good way for a CEO to get fired.

For the interview subjects who did not feel that their hospital was developing cybersecurity capabilities, it was mostly because of high turnover at the C-suite level. That high turnover, in turn, led to constant shifts in strategy that became difficult to navigate as an IS specialist, leaving the organizations more reactive than proactive in developing cybersecurity capabilities. One subject stated the following:

They understand the importance of it, but they don’t understand the amount of finetuning that takes place...We do have support from the C-level. But...there’s a revolving door with the C-level management, so it’s hard to get someone’s ear and hook on to that support.

In general, subjects felt that although the health care industry potentially lagged behind other industries, there was a growing awareness of cyber risk at both board and C-suite levels. This, in turn, gave their teams sufficient institutional power to affect the necessary changes to build cybersecurity capabilities at the administration level of the hospital. However, a hospital has many more employees than just its administrators. Gaining stakeholder alignment with operational staff—doctors and nurses—is much more difficult. This manifested in two ways: (1) open to or at least indiscreet flaunting of IT policies and (2) an underground “procurement” process of medical devices. The latter is discussed in the theme of end point complexity. An example of skirting IT policies was provided by one interviewee:

Say I want to use an ultrasound machine. We have regulatory requirements that require authentication to all of our IT devices. Then your password has to change every 90 days. They just want to use the ultrasound machine. It’s not holding a lot of patient data, they have to memorize their passwords. Then they can’t use their common username and password [because it’s a different device]. They say we’re putting a lot of burden on us, it’s making it difficult to provide seamless patient care. So they create a shared login so that they can provide patient care.

Working against the IT and IS teams was a strong culture of “patient care first.” Many of the medical professionals see cybersecurity standards as a barrier to patient data portability, which increases the paperwork that staff must prepare, increases the likelihood of error rates when patients are new or transferred, decreases the time that they can spend with patients, and, potentially, decreases the ease of collaboration among staff. Gaining buy-in from these staff was more difficult than gaining buy-in from the administration because the medical staff saw less direct impacts to their ability to perform from the consequences of cyber breaches, such as reputational loss or fines. Two interviewees stated the following:

Doctors, that’s a different story...The nature of their work—they have to get patients in and out. They’re probably the least understanding.

When Amazon is asked to open this port, or relax this firewall, the answer is NO, for no one ever. As opposed to some Nobel Laureate who wants us to relax port 1551.

Interview subjects who had gained success with medical staff had done so via three mechanisms:

(1) Direct experience with cyber crisis. One interviewee stated the following:

Millennials and Gen Z—they’ve never had to deal with the old school stuff of… paper to write down information...So, when the technology turns off younger people don’t know what to do...Ransomware [attacks] are wake up calls for them.

Ability to articulate patient harm. Two interviewees stated the following:

You can solve a corporate argument about what is best for the patient. There’s a consistent ability for me to push cybersecurity by focusing it on the patients.

The one common thing is that clinicians will be more accommodating towards taking on security measures that benefit their patients.

And, designing systems such that medical staff never became aware of a way to loosen IT security policy. Two interviewees stated the following:

My hope is that they pay very little attention to it. They shouldn’t have to. Cybersecurity is my job and not theirs.

We have a lot of ransomware attempts, but it’s not a problem. We are unusual in healthcare because no one has local admin rights, so no one saves docs on a computer...and everything is fully recoverable.

Additionally, the complexity of hospitals as organizations can lead to conflicting views about what the primary mission of the hospital is, and as a result, can lead to conflicts over the purpose and acceptable impacts of security policy. The most commonly noted examples were the differences in designing security for research hospitals, teaching hospitals, and safety net hospitals. Teaching hospitals had to ensure that patient data was protected and that residents, attending physicians, and students had sufficient access to patient data to fulfill the hospital’s teaching mission as well. In an example provided by a CIO working at a research hospital, a content filtering service intended to block adult content also impacted the work of a research team studying the effects of pornography on mental health:

I personally believe that hardcore pornography has no purpose on hospital supported devices. What did I do five years ago, I put up internet content filters that prevented people from navigating to pornography. Within five minutes, the director of psychiatry calls to tell me that we have a grant to study pornography in a medical context. It’s really hard in an academic medical center with a 1000 different CEOs—because every academic chair is a CEO.

Many interview subjects referred to their own work as primarily cultural rather than technical, by which they meant working with internal stakeholders to shift the perception of cybersecurity practices as a “nice-to-have,” as illustrated in the following quote:

To me, what that means is that the culture of the organization has to change. Processes are a very strong way of changing the security posture of the organization. It’s not just changing the technology. It’s about the vendors, the workflow you use for onboarding employees, for moving data around the organization. That’s all awareness and training. It’s a real cultural thing that your org has to see security through.

With regards to cybersecurity, this manifests as an increase in end point complexity. While IT attempts to manage hospital’s BYOD policies and network security, they must also contend with an underground procurement process by which clinicians, who have some self-sufficiency with their own department’s budget, can purchase the medical devices that they think best provide patient care. One interviewee stated the following:

Someone has already been working with a vendor, they decide to bring the vendor on site, and decide to work with the vendor. And then, they realize they need the technology, so they call the IT staff. So [IT does] an assessment after the fact, and works with the vendor to implement their solution. It’s a backward process, it’d be nice to look at the vendor before, and go through some references, and have some sort of risk analysis and scoring, where we can say the vendor seems legitimate, and like they have the right solution in place...but that’s not the current state.

**Figure 5:**
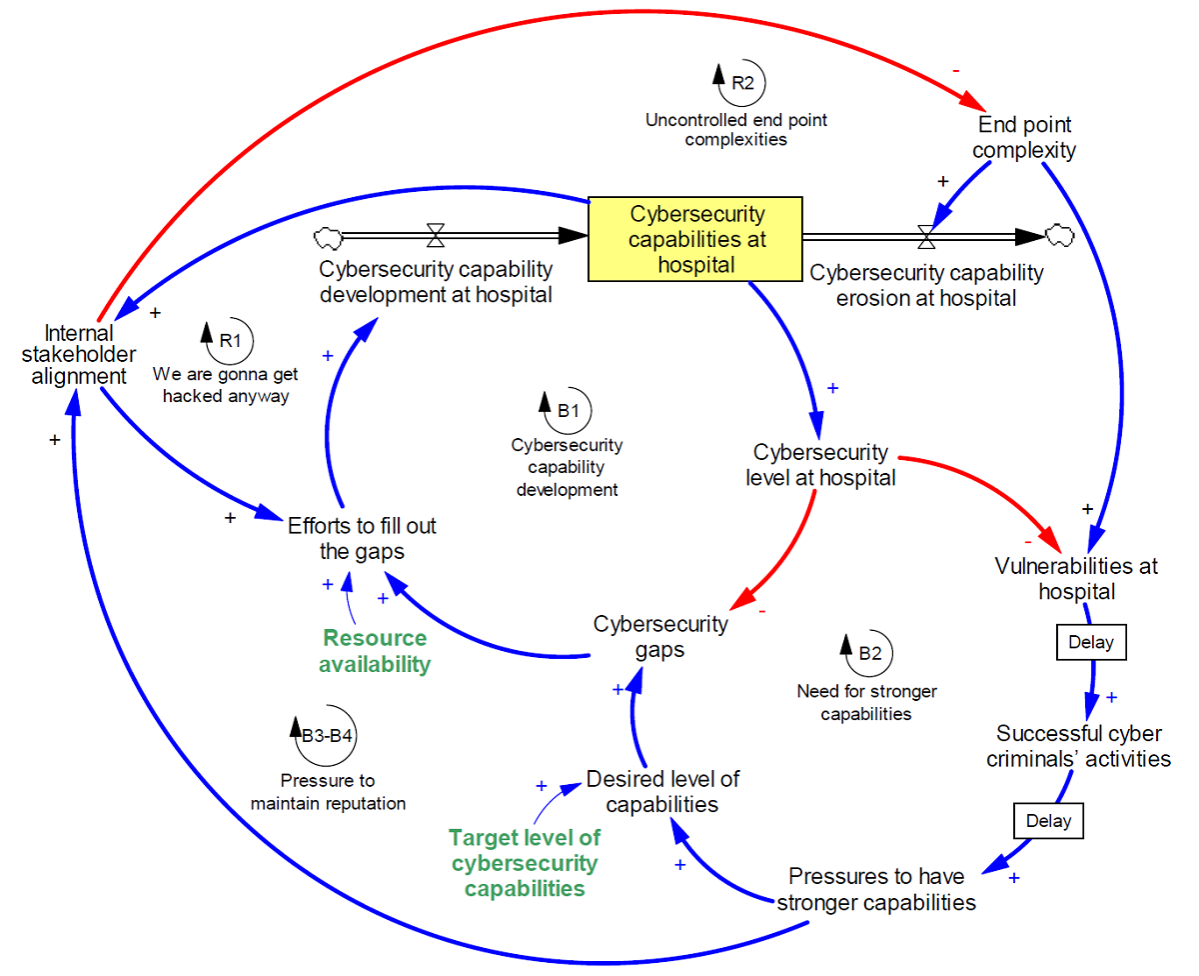
Introduction of internal stakeholder alignment variable.

As a result of all these dynamics, internal stakeholder alignment becomes a highly interconnected variable that influences many other variables and forms two reinforcing loops (R1 and R2) and two balancing loops (B3 and B4; Figure 5). As cybersecurity capabilities decrease and medical staff becomes less aware of the threats they are introducing through new devices or unsafe cyber practices, end point complexity grows. This leads to a reinforcing feedback loop that could result in an explosion of end point complexities. Additionally, if stakeholders do not align with the importance of cybersecurity, they are also more likely to undermine efforts to fill out cybersecurity gaps, reducing cybersecurity capability development, and thus decreasing capabilities. This forms another reinforcing feedback loop, captured by the attitude “We are going to get hacked anyway?” Finally, the pressure to have stronger capabilities manifests within the variable of internal stakeholder alignment and influences how they see efforts to close cybersecurity gaps and end point complexity. This pressure forms two balancing loops, described as the pressure to maintain reputation (see Figure 5 for the addition of this variable and the corresponding feedback loops).

### Cybercriminal Activity

Finally, the dynamics of cybercriminal activity itself is a major driver in this system. Cybercriminals have been highly active in targeting health care organizations, although subjects were split as to whether that was because of an overall increase in cyber activity, or an increase in cyber activity specifically targeted at health care. One subject stated the following:

To be clear, WannaCry was an untargeted attack, whereas Petya and BadRabbit—those appeared to be targeting, although the jury is out, no one has done attribution on that. I think the ransomware risk is real, I think organizations need to take it seriously.

For those who did feel health care was specifically targeted, subjects described three reasons for why there has been increased cybercriminal activity in health care.

If the cybercriminal’s goal is financial, the value of medical data is relatively high compared with other types of data, as illustrated in the following quotes:

If I remember my data correctly, a hospital record costs 20x more than the $10 that you get for someone’s social security number. So hackers aren’t looking for credit cards and bank accounts anymore, they’re looking for medical records which contain a lot more information.

The number, amount, and variety are probably only comparable to a bank that deals with all those transaction volumes. And that this information is required for us to do the most basic activities that we are engaged in.

If the cybercriminal’s goal is ideological or terrorism-related, disrupting the feeling of safety that health care might otherwise inspire is an attractive target to induce fear, as illustrated in the following quote:

I thinkthey do it because it’s scary. Because...when you hit the place the space cares for you when you’re sick, it’s scary...It might have a stronger impact. It makes it to the news faster...If a private organization gets shut down for the day, no one might even know. But if you have to turn away patients from your emergency room because you can’t get your IT up, that’s scary.

And, the health care industry lags other industries in cyber resilience, making them an easy target irrespective of any other qualities, as illustrated in the following quote:

I would aim at a hospital. Healthcare is typically five years behind the power curve. Why not go for soft targets?

In any case, it is clear that health care organizations have been an attractive target recently. Even with an increase in cybersecurity capabilities, the first two reasons for their attractiveness to criminals will remain in place. This overall increasing trend in cybercriminal activities can be incorporated in our model, next to successful cybercriminal activity.

Not specifically included in our model but present nonetheless with our subjects was the feeling, whether substantiated or not, that the motivations of hackers had shifted from being “kids in pajamas” to more malicious organizations: either organized crime or nation-state backed activities. Two subjects stated the following:

The nature of attacks is increasingly sophisticated. It used to be my biggest threat was MIT students. Today, it’s state-sponsored attacks, terrorism, and organized crime. It’s more threats than ever before of a more serious nature.

It’s either economic espionage or geopolitical espionage. Corporate espionage. Basically hacker organizations. It’s gone away from script kiddies, and now it’s nationally sponsored hackers that are actually getting paid to do this.

**Figure 6:**
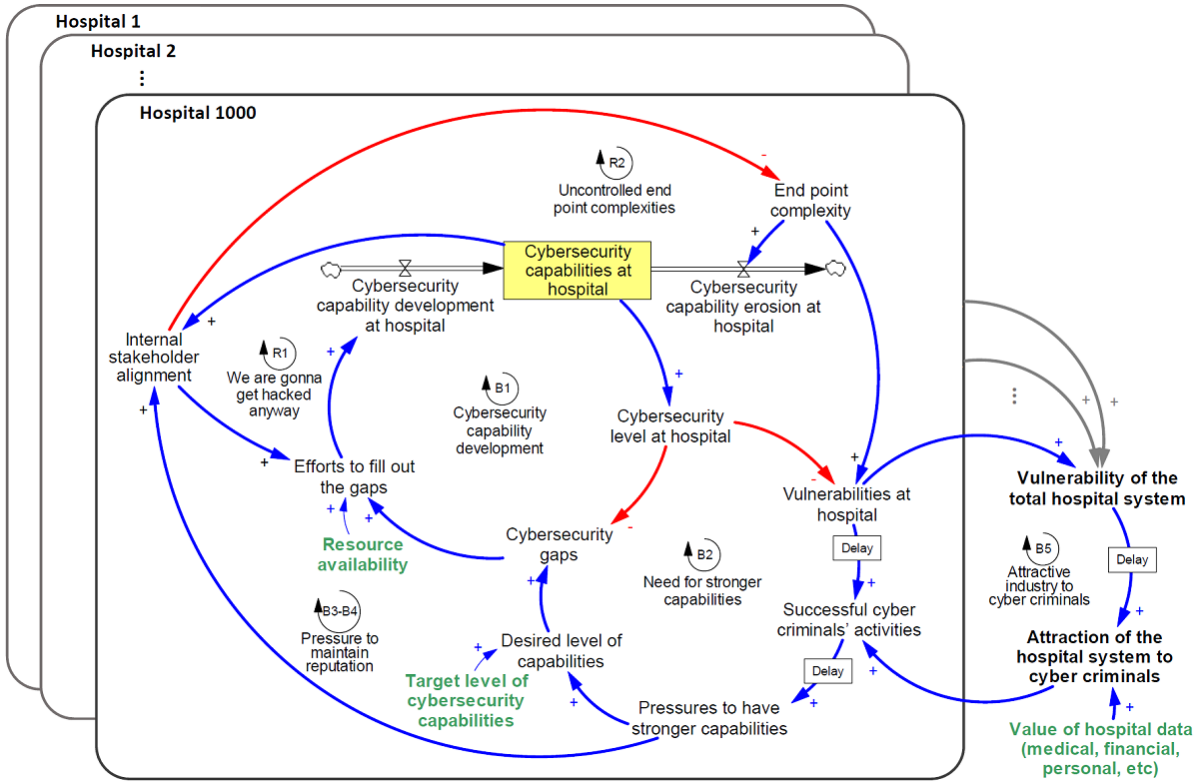
Impact of intravulnerabilities on intervulnerabilities and attractiveness of hospitals system to cybercriminals.

### A Large “System” of Hospital Systems

The mechanisms discussed above manifest themselves not just over a single hospital but over the entire hospital system. The cyber vulnerability of a country’s hospital infrastructure is the result of not just one hospital but rather many hospitals. To represent this in our model, we include 1000 hypothetical hospitals, each with different levels of resource availability and target level of cybersecurity capabilities, and show how the vulnerability of the hospital system, combined with the attractiveness of hospital data would become attractive to cybercriminals (see Figure 6 for this final addition to the modeled system).

## Discussion

### Overview

In the previous section, we used interview data to develop our model. Here, we use simulation analysis to illustrate how the mechanisms in the model might influence each other and can distinguish more resilient hospitals from less resilient ones. We use this to derive potential levers for IT and information security professionals in hospitals to improve cyber resiliency and to identify questions for future potential research.

To study the impact of one parameter in our simulation analyses, we change only that parameter of interest and keep the rest of the model parameters at a hypothetical baseline. The baseline is hypothesized based on resource availability=0.2*,* initial end point complexity=0.8, initial stakeholder alignment=0.2, and desired target level for capabilities=0.5. These parameters are fractions (ie, changing between zero and one representing lowest and highest possible level, respectively). We analyze the effects of a variable at three levels: low (set to 0.1), medium (0.5), and high (0.9). Furthermore, successful cybercriminals’ activities (ie, “vulnerabilities at hospital” × “attraction of the hospital system to cybercriminals”) is assumed to be zero at the beginning of the simulation (Time=0). All simulations are conducted for 60 months.

### Sensitivity of Internal Stakeholder Alignment to Pressures to Improve Capabilities

**Figure 7:**
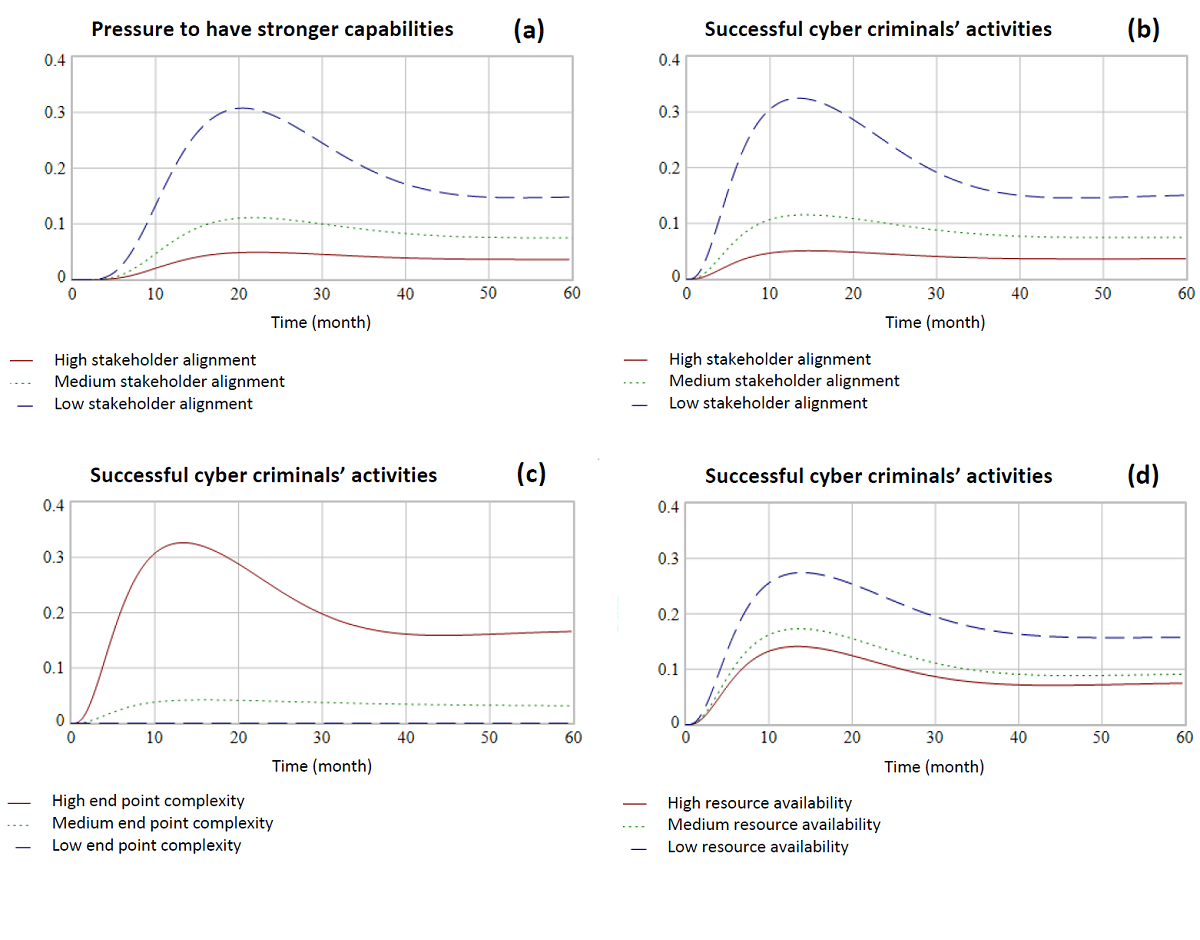
Effects of stakeholder alignment on pressures to improve capabilities over time (a); trends of successful cybercriminals’ activity given the variability in internal stakeholder alignment (b); the variability in end point complexity (c); and the variability in resource availability (d). All y-axes are fractions, changing between zero and one representing lowest and highest possible level, respectively.

Given that there are several causal links between internal stakeholder alignment and the eventual pressure to have stronger capabilities (see Figure 5), we wanted to show how sensitive pressures to improve capabilities is to internal stakeholder alignment. We looked at the behavior of pressure to improve capabilities in a single hospital over time in three scenarios: low, medium, and high; as discussed above (Figure 7).

Figure 7 presents that what might seem to be an initially counterintuitive behavior: Medium to high stakeholder alignment results in low pressure to have stronger capabilities. However, consider that hospitals with medium to high stakeholder alignment likely already have a higher target and desired level of cybersecurity capabilities, the result being that they are less likely to become the victim of a cyberattack. Hospitals with a low stakeholder alignment, however, would be more likely to become the victim of a cyber incident, thus creating pressure to have stronger capabilities. The result of a low stakeholder alignment environment, therefore, would be a high pressure one.

This emphasizes the importance of a CIO’s or CISO’s job in producing stakeholder alignment across the hospital organization.

### Impact of Internal Stakeholder Alignment on Cyberattacks

We also wanted to investigate the direct impact that internal stakeholder alignment has on cyberattacks (“successful cybercriminals’ activity” in the model). We looked at the behavior of successful cybercriminal activity in a single hospital over time in three variations of stakeholder alignment (low, medium, and high; as discussed above; Figure 7).

All three scenarios eventually reach an equilibrium in which the likelihood of a cybercriminal activity is positive. However, both medium and high stakeholder alignment reach this equilibrium without experiencing a period of time during which the likelihood of cybercriminal activity is greatly heightened.

In practical terms, this variability speaks to the importance of the board’s role in cybersecurity governance. If, through governance, the board can create strong stakeholder alignment on the importance of cybersecurity to the organization, this will help minimize the likelihood of cyberattacks.

### Impact of Variability in End Point Complexity on Cyberattacks

Most of our interview subjects suggested that end point complexity was the characteristic that most strongly defined the hospital environment. As a result, we wanted to review to what extent it influenced cyberattacks (“successful cybercriminals’ activity” in the model; see Figure 7). High end point complexity (set to 0.9) had the strongest impact on successful cybercriminal activity relative to moderate (set to 0.5) and low (set to 0.1) end point complexity. With low end point complexity, however, successful cybercriminal activity dropped almost to 0. Although the high-innovative nature of medical environments is associated with the introduction of new technologies that is usually increasing the end point complexity, our results show that minimizing and managing end point complexity across a hospital is an important lever to decrease successful cybercriminal activity. Therefore, cybersecurity professionals should seek for effective interventions to control end point complexity that do not hurt innovation.

### Impact of Variability in Resource Availability on Cyberattacks

Interview subjects were split as to how variability in resource availability affected the hospital system. Some interview subjects felt that a reason that the WannaCry attacks were so harmful to the UK’s National Health Service was that the interconnected nature of their systems raised the resource needs for the maintenance of that large system, and thus lowered the resource availability for cybersecurity initiatives. Others felt that even one hospital among many with fewer available resources for cybersecurity was a threat to the entire infrastructure of health care. Anecdotally, subjects who worked at small outposts of consolidated health care organizations felt protected and safe by their parent organization’s IT and security teams. One subject stated the following:

I think healthcare because of its relatively decentralized nature is particularly vulnerable...There’s consolidation going on in healthcare, there are literally thousands of different organizations across the country.

We investigated the impact of variability in resource availability on cybercriminal activity over time (see Figure 7). Again, we see that higher resource availability decreases the likelihood of successful attack. Interestingly, however, the spread between outcomes for cybercriminal activity is not as wide as it is for variability in internal stakeholder alignment or end point complexity. Additionally, all three scenarios have a ramp-up period before settling into equilibrium. This suggests that given the levers available to them, it is a better use of energy to pursue increasing internal stakeholder alignment and decreasing end point complexity than blindly pursuing higher resource availability.

### Relative Importance of Target Level of Cybersecurity Capabilities and Resource Availability

Most of our interview subjects acknowledged that they operated in a context in which they did not have sufficient resources or were not in full control of the resources at their team’s disposal. This might be for a variety of reasons, including a lack of priority during the hospital’s budgeting process or declining hospital revenues. We use our model to show how the interaction between resource availability and target level of cybersecurity capabilities impacts successful cyber incidents.

**Figure 8:**
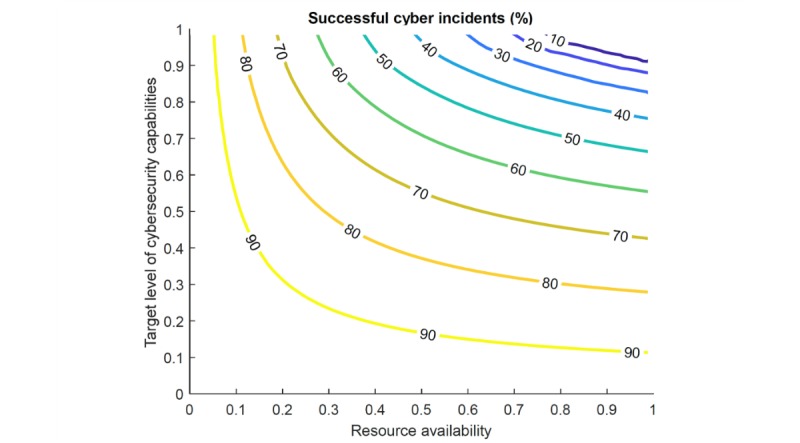
Rate of successful cyber incidents based on target level of cybersecurity capabilities and stakeholder alignment and resource availability.

In Figure 8, we see that at low levels of resource availability on the x-axis (eg, <0.5), even a high target level of cybersecurity capabilities (on the y-axis) does not offer protection from cyber incidents. At high levels of resource availability though, the bigger driver in minimizing successful cyber incidents is the target level of cybersecurity capabilities.

In practice, a hospital that does not have sufficient resources will struggle to develop cybersecurity capabilities and meet a target level of cybersecurity capabilities. They will almost certainly be the victim of a cyberattack, and following the attack, will likely increase resources for cybersecurity (ie, a reactive mode). In our interviews, many of the interviewees felt that their hospital had been at this point a few years ago.

Figure 8 shows that, at high levels of resource availability (eg, >0.5), even at the same target level for cybersecurity capabilities, the likelihood of a successful attack only slightly decreases when more resources are available. However, a CIO or CISO could more significantly decrease the likelihood of a successful attack by simultaneously raising the target level of cybersecurity capabilities for their organization—eg, by designing a prevention plan. Most CIOs or CISOs did attempt to raise their target security levels, typically by publicizing the impacts of cyberattacks on hospital operations at many different levels (board members, C-suite, and staff).

In Multimedia Appendix 1, we provide further analyses on the effects of heterogeneity in resource availability and end point complexity on cybercriminal activities. We also discuss limitations in this study and present suggestions for future research.

### Conclusions

We used interview data to study the complexities of cybersecurity capability development at hospitals. On the basis of the interview data, we developed a system dynamics model and used it to understand how individual hospitals could improve cybersecurity capabilities most effectively. Developing the model demonstrated the existence of three primary levers to improve cybersecurity capabilities available to CIO or CISOs:

Reducing end point complexity: the end point complexity of the hospitals environment is rich with exploitation opportunities for cybercriminals. The tension between decreasing the complexity of this environment and providing excellent patient care is a challenging trade-off. If, however, CIOs and CISOs can decrease the end point complexity of their hospitals, it will have a dramatic impact on decreasing the likelihood of cyberattack. Some of the ways, among many others, that our interviewees achieved the outcome of reducing end point complexity were

- Moving to cloud-hosted services when resource availability was a constraint
- Using technology to detect unauthorized devices on networks
- Maintaining firewalled networks for patients, staff, and medical devices
- Stricter policies on technology procurement

It should also be noted that the end points and what they are connected to are both critical; hence, in addition to the focus on end points, the base architecture needs to be optimized to control the complexity.

Improving internal stakeholder alignment: improving internal stakeholder alignment also reduces the likelihood of cyberattacks. We showed that low internal stakeholder alignment decreases the effectiveness of capability development and increases the erosion of capabilities (by not maintaining them). Our experience shows that soft variables such as stakeholder alignments are often forgotten in cybersecurity management.

Resource availability: finally, while we showed that variability in resource availability did not have the strongest impact on successful cybercriminal activities, we also showed a moderate level of resources is required to have any success in fending off attacks at all. Securing more resources is required to achieve the lowest likelihood of cyberattack, but without internal stakeholder alignment, capabilities are not built and maintained effectively. Furthermore, in the absence of sufficient resources for cybersecurity, setting a high target level of cybersecurity capabilities (beyond those required by policies and regulations) can relatively offset the lack of resources.

Additionally, we used the model to understand what the impact of variability in resource availability within the US health care system has on cybercriminal activity. Our analysis suggests that efforts to homogenize resource availability across hospitals reduce the likelihood of cybercriminal attacks. This effort could be achieved in a few ways. There have already been some efforts to centralize and unify health record data. Using policy as a way to set target levels of cybersecurity capabilities around this health record data could raise the required “floor.” As of today, policy does not specifically address data security, but rather data privacy. Another way would be to work within a single system that assigns resources and set policies to control end point complexity across different hospitals. Although the United States is unlikely to move to the most extreme application of this system (eg, a single player health care), market consolidations have already merged some single hospitals together into a larger system.

Our interview data presents some of the main challenges of cybersecurity capability development at hospitals. Our model also provides an explanatory platform to analyze the complexities development of cybersecurity capabilities in hospitals. For instance, cybersecurity experts believe that resource utilization correlates strongly with infrastructure age: with the increasing arrival of security patches to a hospital IT department, the number of patches increases with the age of systems. These patches need to be tested for their impacts on internal systems, which is a losing endless loop of resource burden. This mechanism can be explained by the general feedback loop B1 in the model, where with the aging systems at a hospital, the cybersecurity level decreases, which in turn requires resources to build capabilities to fill out the cybersecurity gaps.

The potential consequences of cybersecurity risks promoted the Congress to establish the health care industry cybersecurity task force (see [26] for more information), and our study helps complement the work of the task force. It also opens up additional questions for future research, most notably the quantification of the variables introduced in our model. Using this systemic perspective, however, researchers and practitioners can seek to activate or minimize reinforcing processes as their health organization seeks to develop cybersecurity capabilities, thereby improving their resiliency to cyberattacks.

## Appendix

Multimedia Appendix 1 Supplementary information including four sections: (1) interview data summary, (2) effects of heterogeneity in resource availability on cyber-criminal activities, (3) effects of heterogeneity in end point complexity on cyber-criminal activities, and (4) limitations and suggestions for future research.

## Abbreviations

BYOD: bring your own device
CIO: chief information officer
CISO: chief information security officer
COBIT: Control Objectives for Information and Related Technologies
FDA: Food and Drug Administration
HIPAA: Health Insurance Portability and Accountability Act
HITECH: Health Information Technology for Economic and Clinical Health Act
HITRUST: Health Information Trust Alliance
IS: information security
IT: information technology
ITIL: Information Technology Infrastructure Library

## References

[1] Perakslis ED (2014). Cybersecurity in health care. N Engl J Med.

[2] Claunch D, McMillan M (2013). Determining the right level for your IT security investment. Healthc Financ Manage.

[3] Ponemon Institute https://www.ponemon.org/local/upload/file/Sixth%20Annual%20Patient%20Privacy%20%26%20Data%20Security%20Report%20FINAL%206.pdf.

[4] Kruse C, Frederick B, Jacobson T, Monticone D (2017). Cybersecurity in healthcare: a systematic review of modern threats and trends. Technol Health Care.

[5] Smet M (2002). Cost characteristics of hospitals. Soc Sci Med.

[6] Riazul Islam SM, Daehan K, Humaun Kabir M, Hossain M, Kyung-Sup K (2015). The internet of things for health care: a comprehensive survey. IEEE Access.

[7] Brookman J (2015). Protecting privacy in an era of weakening regulation. Harv Law Policy Rev.

[8] Institute of Medicine (2001). Crossing the quality chasm: a new health system for the 21st century. The National Academies Press.

[9] Menachemi N, Burke D, Diana M, Brooks R (2005). Characteristics of hospitals that outsource information system functions. J Healthc Inf Manag.

[10] Menachemi N, Burke D, Diana M, Brooks R (2005). Characteristics of hospitals that outsource information system functions. J Healthc Inf Manag.

[11] Angst CE, Block Es, D’Arcy J, Kelley K (2017). When do IT security investments matter? accounting for the influence of institutional factors in the context of healthcare data breaches. MISQ.

[12] - (2015). 37% of hospitals perform cybersecurity incident response exercises annually. Trustee.

[13] Park W, Seo S, Son S, Lee M, Kim S, Choi E, Bang J, Kim Y, Kim O (2010). Analysis of information security management systems at 5 domestic hospitals with more than 500 beds. Healthc Inform Res.

[14] Namoğlu N, Ulgen Y (2013). Network security vulnerabilities and personal privacy issues in Healthcare Information Systems: a case study in a private hospital in Turkey. Stud Health Technol Inform.

[15] Zarei J, Sadoughi F (2016). Information security risk management for computerized health information systems in hospitals: a case study of Iran. Risk Manag Healthc Policy.

[16] Laabes EP, Nyango DD, Ayedima MM, Ladep NG (2010). Physician use of updated anti-virus software in a tertiary Nigerian hospital. Niger J Med.

[17] Humaidi N, Balakrishnan V (2018). Indirect effect of management support on users' compliance behaviour towards information security policies. Health Inf Manag.

[18] Sittig DF, Singh H (2016). A socio-technical approach to preventing, mitigating, and recovering from ransomware attacks. Appl Clin Inform.

[19] Harrell M, Bradley M (2009). Data collection methods: semi-structured interviews and focus groups. RAND Corporation.

[20] Patton M (2002). Qualitative research and evaluation methods.

[21] Luna-Reyes LF, Andersen DL (2004). Collecting and analyzing qualitative data for system dynamics: methods and models. Syst Dyn Rev.

[22] Jalali M, Siegel M, Madnick S (2017). Arxiv.

[23] Jalali M, Kaiser J, Siegel M, Madnick S (2017). SSRN.

[24] Jalali M, Rahmandad H, Bullock S, Ammerman A (2017). Dynamics of implementation and maintenance of organizational health interventions. IJERPH.

[25] Jalali MS, Ashouri A, Herrera-Restrepo O, Zhang H (2016). Information diffusion through social networks: the case of an online petition. Expert Syst Appl.

[26] Jarrett MP (2017). Cybersecurity—a serious patient care concern. JAMA.

