# Supplementary material for: Cybersecurity in Hospitals: A Systematic, Organizational Perspective
Source: J Med Internet Res. 2018 May 28;20(5):e10059. doi: 10.2196/10059 (PMC5996174; doi:10.2196/10059)
Supplement: Multimedia Appendix 1 [file jmir_v20i5e10059_app1.pdf]

Multimedia Appendix 1

Supplementary information

for

Cybersecurity in Hospitals: a Systematic, Organizational Perspective

Mohammad S. Jalali, PhD; Jessica P. Kaiser, MBA
MIT Sloan School of Management, Cambridge, MA, United States

Contents

Interview Data Summary 1
Effects of Heterogeneity in Resource Availability on Cyber-criminal Activities 1
Effects of Heterogeneity in End point Complexity on Cyber-criminal Activities 3
Limitations and Suggestions for Future Research 4

Interview Data Summary

Table 1. Interview data summary

**Level**		**Size**		**Urbanicity**		**Profit Orientation**	
C-Level	12 (63%)	<149 beds	3 (16%)	Rural	2 (10%)	For-profit	10 (53%)
IS Specialist	4 (21%)	>150 & <=1k beds	8 (42%)	City <500k	3 (16%)	Not-for-profit	9 (47%)
Vendor/Consultant	3 (16%)	>=1k beds	4 (21%)	City >=500k	10 (53%)		
		n/a	4 (21%)	Mixed	4 (21%)		

IS: Information Security

Effects of Heterogeneity in Resource Availability on Cyber-criminal Activities

As discussed in the article, the cyber vulnerability of a country's hospital infrastructure is the result of many hospitals. Each hospital may have dramatically different cyber capabilities. The U.S. hospital system is market-based and thus is very decentralized and heterogeneous with regards to its cyber capabilities.

By layering our model for a single hospital into a larger model with 1,000 hospitals, we can better understand the cyber resiliency of the entire ecosystem. In Figure S1, we drew the resource availability for these hospitals from a random uniform distribution $u(0,1)$. This represents a healthcare system like that of the U.S., which is very heterogeneous. In Figure S2, however, we drew the resource availability for 1,000 hospitals from a random normal distribution $N(0.5, 0.05)$, which has low variability and represents a homogenous hospital system more like the UK's NHS. It could also be considered to represent a smaller hospital system that has outsourced its cybersecurity to a larger entity. Both distributions have the same mean with regards to resource availability.

Figure S1. Sensitivity of successful cyber-criminals' activity to resource availability in a heterogeneous setting.

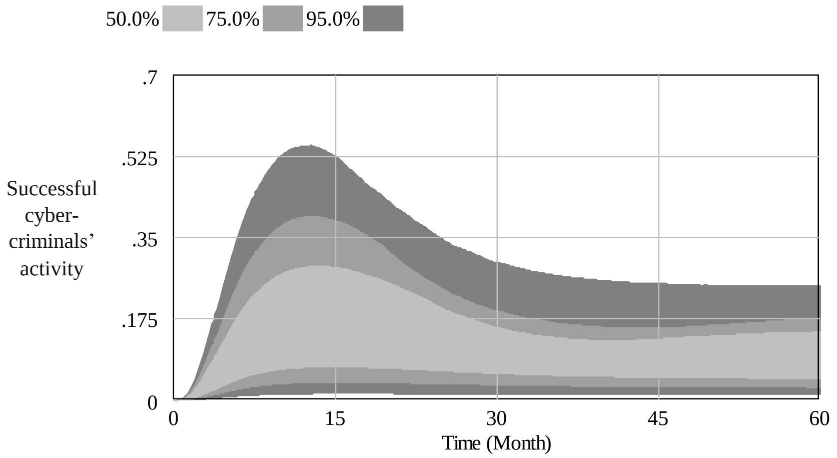

Figure S2. Sensitivity of successful cyber-criminals' activity to resource availability in a homogeneous setting.

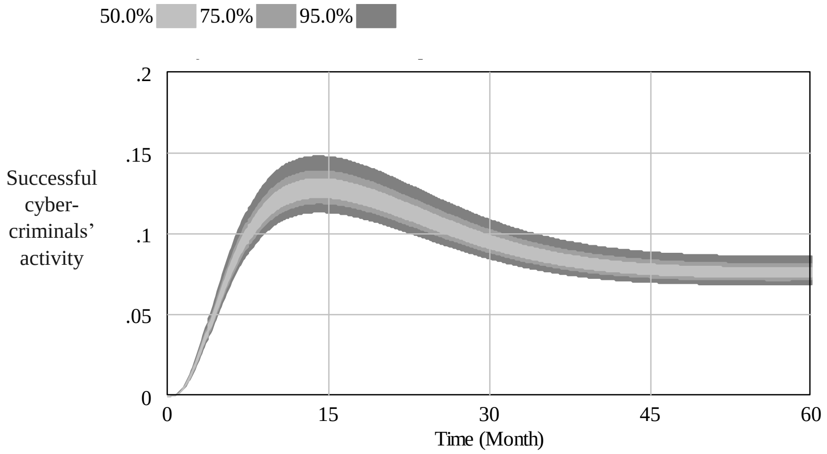

Unsurprisingly, cyber-criminal activities in the heterogeneous setting have a wider variance. Note, however, the scale of successful cyber-criminal activities: in a homogeneous setting, not only is there less variance, but even the hospitals with the highest likelihood of cyber-criminal activities have lower chances than many of the hospitals in the heterogeneous settings.

This analysis lends support to smaller hospitals' decision to outsource cybersecurity. By making their resource availability more uniform through outsourcing, they reduce the variability for other hospitals in the U.S., thus decreasing the likelihood of successful cyber-criminal activities across the system.

Effects of Heterogeneity in End point Complexity on Cyber-criminal Activities

Heterogeneity in end point complexity does not influence successful cyber-criminal activities in the same way as heterogeneity in resource availability (discussed above). On the one hand, in a heterogeneous setting (where end point complexity is drawn from a random uniform distribution $u(0,1)$), there is wider variability in successful cyber-criminal activities, just as there is with heterogeneity in resource availability. However, only a moderate percentage of hospitals have successful cyber-criminal activities <.1. See Figure S3.

Figure S3. Sensitivity of successful cyber-criminals' activity to end point complexity in a heterogeneous setting.

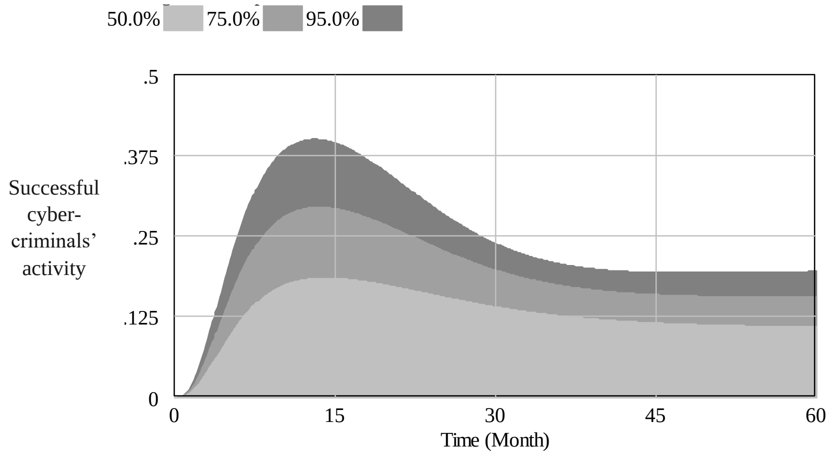

In a homogeneous setting with high end point complexity (drawn from a random normal distribution $N(0.75, 0.05)$), while variability decreases, the mean likelihood of successful cyber-criminal activities increases. See Figure S4. A bigger effect on the likelihood of successful cyber-criminal activities comes from decreasing the mean of end point complexity from "high" (0.75) to "moderate" (0.5). See Figure S5. This suggests that efforts by individual hospitals to reduce end point complexity help reduce cyber vulnerabilities.

Figure S4. Sensitivity of successful cyber-criminals' activity to end point complexity in a homogeneous "high" setting.

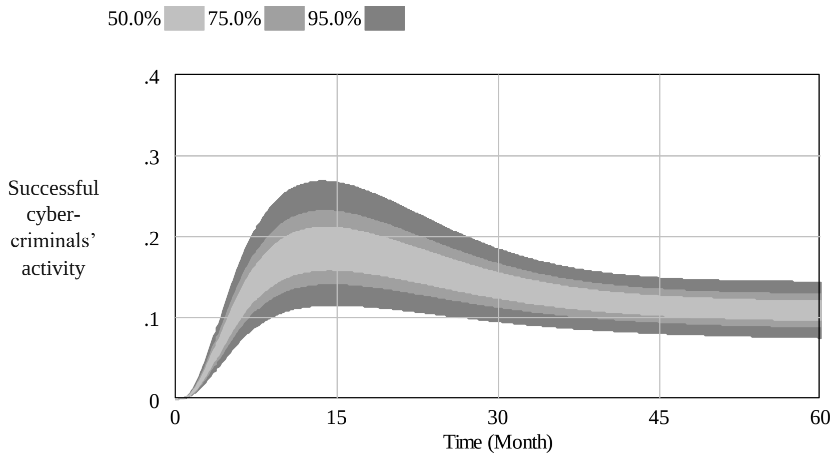

Figure S5. Sensitivity of successful cyber-criminals' activity to end point complexity in a homogeneous moderate setting.

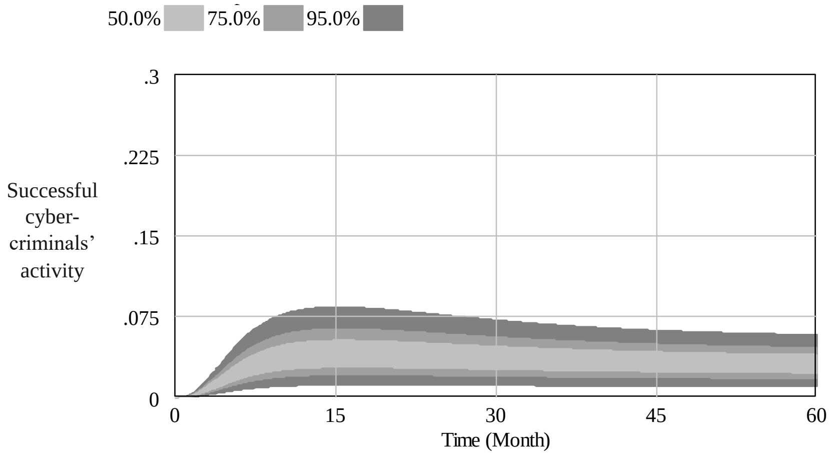

While we argue that high end point variability can increase the likelihood of attack, it should be noted that we do not intend to recommend reducing the variation of end point complexity, as a homogenous system might be easier for cybercriminals to target. Future research must be done to study the tradeoffs in the heterogeneity of end point complexity in more depth.

Limitations and Suggestions for Future Research

We should note that the purpose of this research is to build theory and not to predict. In the absence of detailed quantitative data for cybersecurity in hospitals, one should be cautious about seeking specific operational advice from our model.

Also, a limitation of this study is that it does not take into account the cost of closing cybersecurity gaps. Implicitly, if CISOs had unlimited resources available, cost of market solutions would not be a factor. In our interviews, rather than focus on the cost of solutions, our interviewees merely reflected whether they felt they had the budget to buy them. In practice of cybersecurity capability development, cost is a more important factor. Our intention in developing this model, however, was to analyze the dynamics and "what if" scenarios, rather than "how to" scenarios. Future research might incorporate cost into this model so that information security managers would play with "how" scenarios—e.g., how to effectively control end point complexity that does not hurt innovation. Future studies could also add more external stakeholders to the model, especially those providing IT services. Additionally, this model could be improved upon by quantifying all variables more rigorously.
